# Automatically tracking neurons in a moving and deforming brain

**DOI:** 10.1371/journal.pcbi.1005517

**Published:** 2017-05-18

**Authors:** Jeffrey P. Nguyen, Ashley N. Linder, George S. Plummer, Joshua W. Shaevitz, Andrew M. Leifer

**Affiliations:** 1 Department of Physics, Princeton University, Princeton, New Jersey, United States of America; 2 Princeton Neuroscience Institute, Princeton University, Princeton, New Jersey, United States of America; 3 Lewis-Sigler Institute for Integrative Genomics, Princeton University, Princeton, New Jersey, United States of America; Northwestern University, UNITED STATES

## Abstract

Advances in optical neuroimaging techniques now allow neural activity to be recorded with cellular resolution in awake and behaving animals. Brain motion in these recordings pose a unique challenge. The location of individual neurons must be tracked in 3D over time to accurately extract single neuron activity traces. Recordings from small invertebrates like *C. elegans* are especially challenging because they undergo very large brain motion and deformation during animal movement. Here we present an automated computer vision pipeline to reliably track populations of neurons with single neuron resolution in the brain of a freely moving *C. elegans* undergoing large motion and deformation. 3D volumetric fluorescent images of the animal’s brain are straightened, aligned and registered, and the locations of neurons in the images are found via segmentation. Each neuron is then assigned an identity using a new time-independent machine-learning approach we call Neuron Registration Vector Encoding. In this approach, non-rigid point-set registration is used to match each segmented neuron in each volume with a set of reference volumes taken from throughout the recording. The way each neuron matches with the references defines a feature vector which is clustered to assign an identity to each neuron in each volume. Finally, thin-plate spline interpolation is used to correct errors in segmentation and check consistency of assigned identities. The Neuron Registration Vector Encoding approach proposed here is uniquely well suited for tracking neurons in brains undergoing large deformations. When applied to whole-brain calcium imaging recordings in freely moving *C. elegans*, this analysis pipeline located 156 neurons for the duration of an 8 minute recording and consistently found more neurons more quickly than manual or semi-automated approaches.

## Author summary

Computer algorithms for identifying and tracking neurons in images of a brain have struggled to keep pace with rapid advances in neuroimaging. In small transparent organism like the nematode *C. elegans*, it is now possible to record neural activity from all of the neurons in the animal’s head with single-cell resolution as it crawls. A critical challenge is to identify and track each individual neuron as the brain moves and bends. Previous methods required large amounts of manual human annotation. In this work, we present a fully automated algorithm for neuron segmentation and tracking in freely behaving *C. elegans*. Our approach uses non-rigid point-set registration to construct feature vectors describing the location of each neuron relative to other neurons and other volumes in the recording. Then we cluster feature vectors in a time-independent fashion to track neurons through time. This new approach works very well when compared to a human.

## Introduction

Optical neural imaging has ushered in a new frontier in neuroscience that seeks to understand how neural activity generates animal behavior by recording from large populations of neurons at cellular resolution in awake and behaving animals. Population recordings have now been used to elucidate mechanisms behind zebra finch song production [1], spatial encoding in mice [2], and limb movement in primates [3]. When applied to small transparent organisms, like *Caenorhabditis elegans* [4], *Drosophila* [5], and zebrafish [6], nearly every neuron in the brain can be recorded, permitting the study of whole brain neural dynamics at cellular resolution.

Methods for segmenting and tracking neurons have struggled to keep up as new imaging technologies now record from more neurons over longer times in environments with greater motion. Accounting for brain motion in particular has become a major challenge, especially in recordings of unrestrained animals. Brains in motion undergo translations and deformations in 3D that make robust tracking of individual neurons very difficult. The problem is compounded in invertebrates like *C. elegans* where the head of the animal is flexible and deforms greatly. If left unaccounted for, brain motion not only prevents tracking of neurons, but it can also introduce artifacts that mask the true neural signal. In this work we propose an automated approach to segment and track neurons in the presence of dramatic brain motion and deformation. Our approach is optimized for calcium imaging in unrestrained *C. elegans*.

Neural activity can be imaged optically with the use of genetically encoded calcium sensitive fluorescent indicators, such as GCaMP6s used in this work [7]. Historically calcium imaging was often conducted in head-fixed or anesthetized animals to avoid challenges involved with imaging moving samples [4, 8, 9]. Recently, however, whole-brain imaging was demonstrated in freely behaving *C. elegans* [10, 11]. *C. elegans* are a small transparent nematode, approximately 1mm in length, with a compact nervous system of only 302 neurons. About half of the neurons are located in the animal’s head, which we refer to as its brain.

Analyzing fluorescent images of moving and deforming brains requires algorithms to detect neurons across time and extract fluorescent signals in 3D. Automated methods exist for segmenting and tracking fluorescently labeled cells during *C. elegans* embryogenesis [12], and semi-automated methods are even able to track specific cells during embryo motion [13], but to our knowledge these methods are not suitable for tracking neurons in adults. Generally, several strategies exist for tracking neurons in volumetric recordings. One approach is to find correspondences between neuron positions in consecutive time points, for example, by applying a distance minimization, and then stitching these correspondences together through time [14]. This type of time-dependent tracking requires that neuron displacements for each time step are less than the distance between neighboring neurons, and that the neurons remain identifiable at all times. If these requirements break down, even for only a few time points, errors can quickly accumulate. Other common methods, like independent component analysis (ICA) [15] are also exquisitely sensitive to motion and as a result they have not been successfully applied to recordings with large brain deformations.

Large inter-volume motion arises when the recorded image volume acquisition rate is too low compared to animal motion. Unfortunately, large inter-volume brain motion is likely to be a prominent feature of whole-brain recordings of moving brains for the foreseeable future. In all modern imaging approaches there is a fundamental tradeoff between the following attributes: acquisition rate (temporal resolution), spatial resolution, signal to noise, and the spatial extent of the recording. As recordings seek to capture larger brain regions at single cell resolution, they necessarily compromise on temporal resolution. For example, whole brain imaging in freely moving *C. elegans* has only been demonstrated at slow acquisition rates because of the requirements to scan the entire brain volume and expose each slice for sufficiently long time. At these rates, a significant amount of motion is present between image planes within a single brain volume. Similarly, large brain motions also remain between sequential volumes. Neurons can move the entire width of the worm’s head between sequential volumes when recording at 6 brain-volumes per second, as in [10]. In addition to motion, the brain also bends and deforms as it moves. Such changes to the brain’s conformation greatly alter the pattern of neuron positions making constellations of neurons difficult to compare across time.

To track neurons in the presence of this motion, previous work that measured neural activity in freely moving *C. elegans* utilized semi-automated methods that required human proof reading or manual annotation to validate each and every neuron-time point [10, 11]. This level of manual annotation becomes impractical as the length of recordings and the number of neurons increases. For example, 10 minutes of recorded neural activity from [10], had over 360,000 neuron time points and required over 200 person-hours of proofreading and manual annotation. Here, we introduce a new time-independent algorithm that uses machine learning to automatically segment and track all neurons in the head of a freely moving animal without the need for manual annotation or proofreading. We call this technique Neuron Registration Vector Encoding, and we use it to extract neural signals in unrestrained *C. elegans* expressing the calcium indicator GCaMP6s and the fluorescent label RFP.

## Results

### Overview of neuron tracking analysis

**Fig 1:**
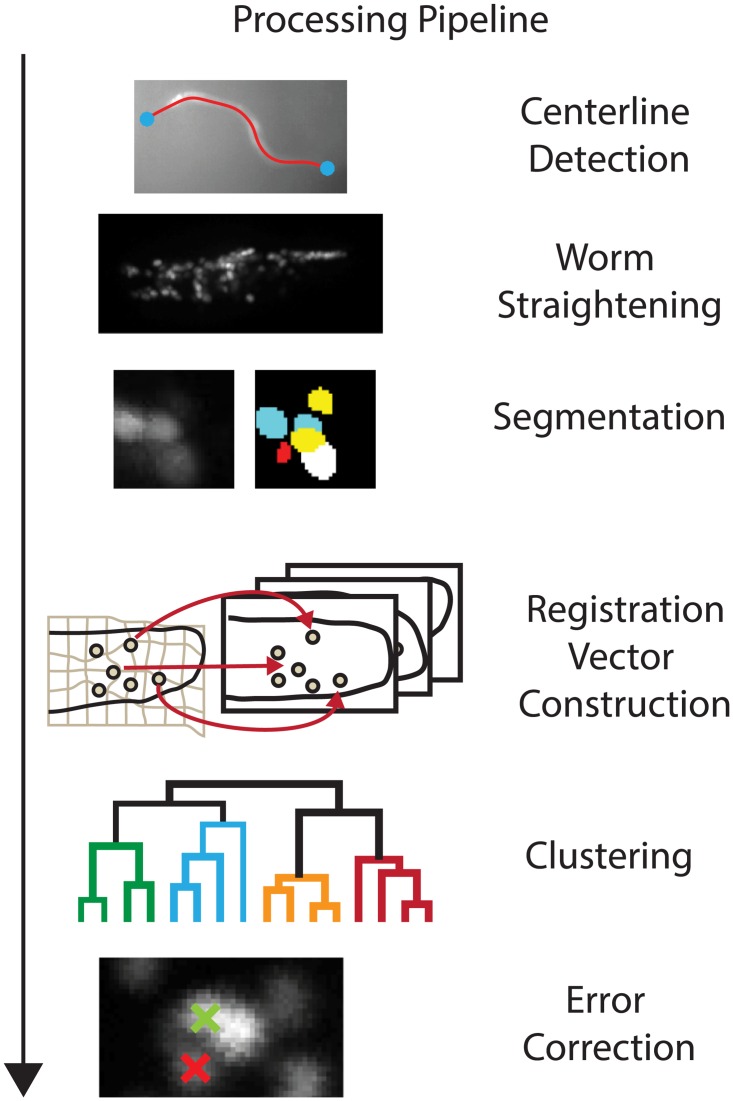
Schematic of analysis pipeline to segment and track neurons through time and extract their neural activity in a deforming brain. Neurons are labeled with calcium insensitive red fluorescent proteins, RFP, and calcium sensitive green fluorescent proteins, GCaMP. Videos of the animal’s behavior and volumetric fluorescent images of the animal’s brain serve as input to the pipeline. The algorithm detects all neurons in the head and produces tracks of the neural activity across time as the animal moves.

We introduce a method to track over 100 neurons in the brain of a freely moving *C. elegans*. The analysis pipeline is made of five modules and an overview is shown in Fig 1. The first three modules, “Centerline Detection,” “Straightening” and “Segmentation,” collectively assemble the individually recorded planes into a sequence of 3D volumes and identify each neuron’s location in each volume. The next two modules, “Registration Vector Construction” and “Clustering,” form the core of the method and represent a significant advance over previous approaches. Collectively, these two modules are called “Neuron Registration Vector Encoding.” The “Registration Vector Construction” module leverages information from across the entire recording in a time-independent way to generate feature vectors that characterize every neuron at every time point in relation to a repertoire of brain confirmations. The “Clustering” module then clusters these feature vectors to assign a consistent identity to each neuron across the entire recording. A final module corrects for errors that can arise from segmentation or assignment. The implementation and results of this approach are described below.

### Recording of whole-brain calcium activity and body posture in moving animal

Worms expressing the calcium indicator GCaMP6s and a calcium-insensitive fluorescent protein RFP in the nuclei of all neurons were imaged during unrestrained behavior in a custom 3D tracking microscope, as described in [10]. Only signals close to the cell nuclei are measured. Two recordings are presented in this work: a new 8 minute recording of an animal of strain AML32 and a previously reported 4 minute recording of strain AML14 first described in [10].

**Fig 2:**
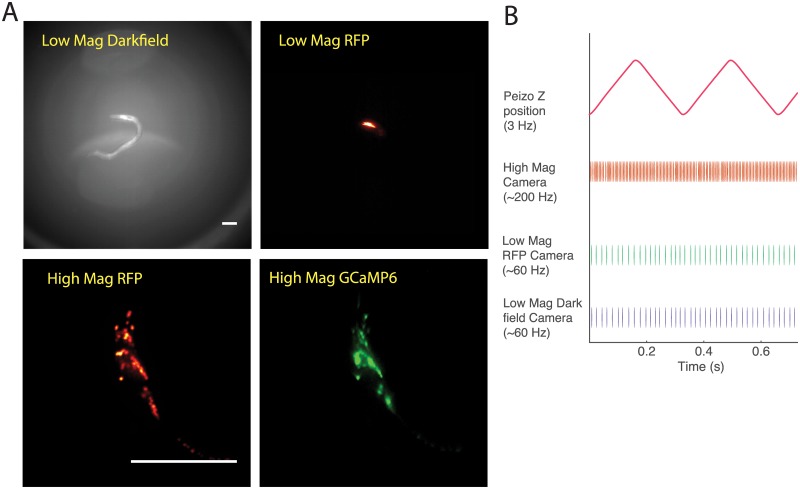
Input to the pipeline. (A) Example images from all four video feeds from our imaging system. Both scale bars are 100*μ*m. Fluorescent images are shown with false coloring. (B)A schematic illustrating the timings from all the devices that run in open loop in our imaging setup. The camera that collects high magnification images captures at 200Hz. The two low magnification images capture at 60Hz, and the focal plane moves up and down in a 3 Hz triangle wave. The cameras are synchronized post-hoc using light flashes and each image is assigned a timestamp on a common timeline.

The signal of interest in both recordings is the green fluorescence intensity from GCaMP6s in each neuron. Red fluorescence from the RFP protein serves as a reference for locating and tracking the neurons. The microscope provides four raw image streams that serve as inputs for our neural tracking pipeline, seen in Fig 2A. They are: (1) low-magnification dark-field images of the animal’s body posture (2) low-magnification fluorescent images of the animal’s brain (3) high-magnification green fluorescent images of single optical slices of the brain showing GCaMP6s activity and (4) high-magnification red fluorescent images of single optical slices of the brain showing the location of RFP. The animal’s brain is kept centered in the field of view by realtime feedback loops that adjust a motorized stage to compensate for the animal’s crawling. To acquire volumetric information, the high magnification imaging plane scans back and forth along the axial dimension, *z*, at 3 Hz as shown in Fig 2B, acquiring roughly 33 optical slices per volume, sequentially, for 6 brain-volumes per second. The animal’s continuous motion causes each volume to be arbitrarily sheared. Although the image streams operate at different volume acquisition rates and on different clocks, they are later synchronized by flashes of light that are simultaneously visible to all cameras. Each image in each stream is given a timestamp on a common timeline for analysis. Each of the four imaging streams are spatially aligned to each other in software using affine transformations found by imaging fluorescent beads. An example of the high magnification RFP recording is shown in S1 Movie.

### Centerline detection and gross brain alignment

The animal’s posture contains information about the brain’s orientation and about any deformations arising from the animal’s side-to-side head swings. The first step of the pipeline is to extract the centerline that describes the animal’s posture. Centerline detection in *C. elegans* is an active field of research. Most algorithms use intensity thresholds to detect the worm’s body and then use binary image operations to extract a centerline [16–18]. Here we use an open active contour approach [19, 20] to extract the centerline from dark field images with modifications to account for cases when the worm’s body crosses over itself as occurs during so-called “Omega Turns.” In principle any method, automated or otherwise, that detects the centerlines should be sufficient. At rare times where the worm is coiled and the head position and orientation cannot be determined automatically, the head and the tail of the worm are manually identified.

**Fig 3:**
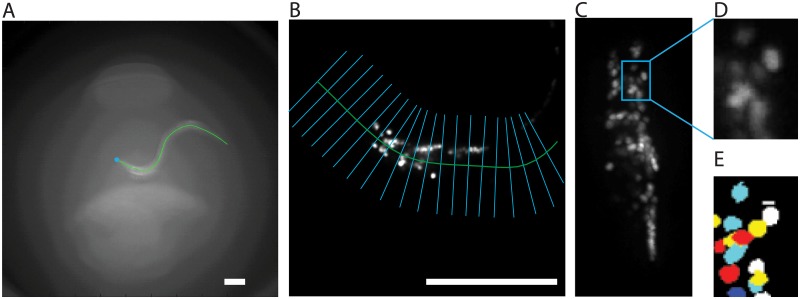
Straightening and segmentation. (A) Centerlines are detected from the low magnification dark field images. The centerline is shown in green and the tip of the worm’s head is indicated by a blue dot. (B) The centerline found from the low magnification image is overlaid on the high magnification RFP images. The lines normal to the centerline, shown in blue, are used to straighten the image. All scale bars are 100 *μ*m. (C) A maximum intensity projection of the straightened volume is shown. Individual neuronal nuclei are shown (D) before and (E) after segmentation.

The animal’s centerline allows us to correct for gross changes in the worm’s position, orientation, and conformation (Fig 3a). We use the centerlines determined by the low magnification behavior images to straighten the high magnification images of the worm’s brain. An affine transform must be applied to the centerline coordinates to transform them from the dark field coordinate system into the coordinate system of the high magnification images. Each image slice of the worm brain is straightened independently to account for motion within a single volume. The behavior images are taken at a lower acquisition rate than the high magnification brain images, so a linear interpolation is used to obtain a centerline for each slice of the brain volume. In each slice, we find the tangent and normal vectors at every point of the centerline (Fig 3b). The points are interpolated with a single pixel spacing along the centerline to preserve the resolution of the image. The image intensities along each of the normal directions are interpolated and the slices are stacked to produce a straightened image in each slice (Fig 3c). In the new coordinate system, the orientation of the animal is fixed and the gross deformations from the worm’s bending are suppressed. More subtle motion and deformation, however, remains.

We further reduce shearing between slices using standard video stabilization techniques [21]. Specifically, bright-intensity peaks in the images are tracked between neighboring image slices. The coordinates of these peaks are used to calculate the affine transformations between neighboring slices of the volume using least squares. All slices are registered to the middle slice by applying these transformations sequentially throughout the volume. Each slice would undergo transformations for every slice in between it and the middle slice to correct shear throughout the volume. A final rigid translation is required to align each volume to the first volume of the recording. The translations are found by finding an offset that maximizes the cross-correlation between each volume and the initial volume.

A video of straightening is shown in S1 Movie. Straightened images are used for the remaining steps of the analysis pipeline. Only the final measurement of fluorescence intensity is performed in the original unstraightened coordinated system.

### Segmentation

Before neuron identities can be matched across time, we must first segment the individual neurons within a volume to recovers each neuron’s size, location, and brightness (Fig 3d and 3e). Many algorithms have been developed to segment neurons in a dense region [22, 23]. We segment the neurons by finding volumes of curvature in fluorescence intensity in the straigthened volumes. After an initial smoothing, we compute the 3D Hessian matrix at each point in space and threshold for points where all of the three eigenvalues of the Hessian matrix are negative. This process selects for regions around intensity peaks in three dimensions. In order to further divide regions into objects that are more likely to represent neurons, we use a watershed separation on the distance transform of the thresholded image. The distance transform is found by replacing each thresholded pixel with the Euclidean distance between it and the closest zero pixel in the thresholded image. This approach is sufficient to segment most neurons. Occasionally neurons are missed or two neurons are incorrectly merged together. These occasional errors are corrected automatically later in the pipeline.

### Neuron registration vector construction

**Fig 4:**
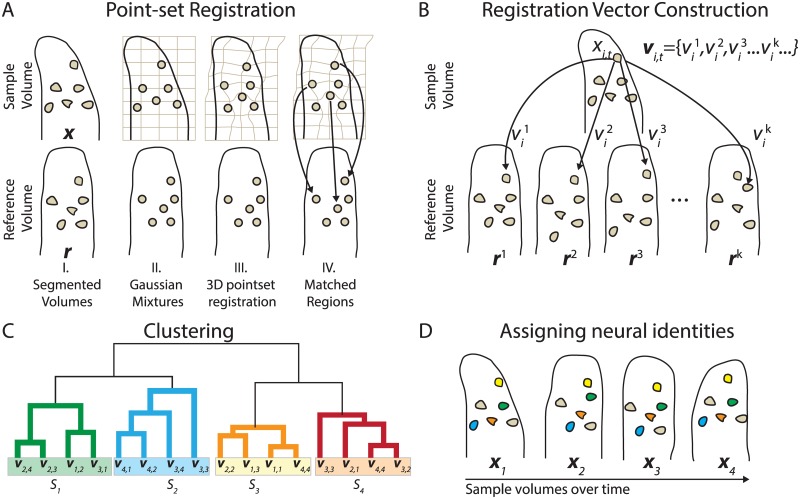
Schematic of Neuron Registration Vector Encoding. (A) The registration between a sample volume and a single reference volume is done in several steps. I. The image is segmented into regions corresponding to each of the neurons. II. The image is represented as a Gaussian mixture, with a single Gaussian for each segmented region. The amplitude and the standard deviation of the Gaussians are derived from the brightness and the size of the segmented regions. III. Non-rigid point-set registration is then used to deform the sample points to best overlap the reference point-set. IV. Neurons from the sample and the reference point-sets are paired by minimizing distances between neurons. (B) Neuron registration vectors are constructed by assigning a feature vector **v**_*i*,*t*_ to each neuron *x*_*i*,*t*_ in a sample volume **x**_*t*_ by performing the registration between the sample volume and a set of 300 reference volumes, each denoted by **r**^*k*^. Each registration of the neuron results in a neuron match, $v_{i}^{k}$, and the set of matches becomes the feature vector **v**_*i*,*t*_. (C) Vectors from all neuron-times are clustered into similar groups in a two step process: Hierarchical clustering (illustrated in the figure) is performed on a subset of neurons to define clusters, each of which is given a label *S*_*n*_. Then each feature vector **v**_*i*,*t*_ is assigned to a cluster based on a distance metric (not illustrated). (D) The clustering of the feature vectors shown in (C) assigns an identity to each of the neurons in every volume. This allows us to track the neurons across different volumes of the recording.

Extracting neural signals requires the ability to match neurons found at different time points. Even after gross alignment and straightening, neurons in our images are still subject to local nonlinear deformations and there is significant movement of neurons between volumes. This remaining motion and deformation is clearly visible, for example, in S1 Movie. Rather than tracking neurons sequentially in time, the neurons in each volume are characterized based on how they match to neurons in a set of reference volumes. Our algorithm compares constellations of neurons in one volume to unannotated reference volumes and assigns correspondences or “matches” between the neurons in the sample and each reference volume. We modified a point-set registration algorithm developed by Jian and Vemuri [24] to do this (Fig 4a). The registration algorithm represents two point-sets, a sample point-set denoted by **X** = {**x**_*i*_} and a reference point-set indicated by **R** = {**r**_*i*_}, as Gaussian mixtures and then attempts to register them by deforming space to minimize the distance between the two mixtures. In their implementation, each point is modeled by a 3D Gaussian with uniform covariance. Since we are matching images of neurons rather than just points, we can use the additional information from the size and brightness of each neuron. We add this information to the representation of each neuron by adjusting the amplitude and standard deviation of the Gaussians. The Gaussian mixture representation of an image is given by,

$$\begin{array}{c} f\left(\xi ,X\right)=\underset{i}{\sum }A_{i}\text{exp}\left(-\frac{{\left\|\xi -x_{i}\right\|}^{2}}{2{\left(\lambda \sigma_{i}\right)}^{2}}\right), \end{array}$$ (1)

where *A*_*i*_, **x**_*i*_, and *σ*_*i*_ are the amplitude, mean, and standard deviation of the *i*-th Gaussian. These parameters are derived from the brightness, centroid, and size of the segmented neuron, while ***ξ*** is the 3D spatial coordinate. A scale factor *λ* is added to the standard deviation to scale the size of each Gaussian. This will be used later during gradient descent. The sample constellation of neurons is then represented by the Gaussian mixture *f*(***ξ***, **X**). Similarly, the reference constellation’s own neurons are represented as a *f*(***ξ***, **R**).

To match a sample constellation of neurons **X** with a reference constellation of neurons **R**, we use the non rigid transformation $u:I\;R^{3}\mapsto I\;R^{3}$. The transformation maps **X** to *u*[**X**] such that the *L*_2_ distance between *f*(***ξ***, *u*[**X**]) and *f*(***ξ***, **R**) is minimized with some constraint on the amount of deformation. This can be written as an energy minimization problem, with the energy of the transformation, *E*(*u*), written as

$$\begin{array}{c} E\left(u\right)=\int {\left[f(\xi ,u[X])-f(\xi ,R)\right]}^{2}d\xi +E_{\text{Deformation}}\left(u\right). \end{array}$$ (2)

Note that the point-sets **X** and **R** are allowed to have different numbers of points. We model the deformations as a thin-plate spline (TPS). The TPS transformation equations and resulting form of *E*_Deformation_(*u*) are shown in the methods. The minimization of *E* is found by gradient descent. Working with Gaussian mixtures as opposed to the original images allows us to model the deformations and analytically compute the gradients of Eq 2 making gradient descent more efficient. The gradient descent approach used here is similar to that outlined by Jian and Vemuri [25]. Since the energy landscape has many local minima, we initially chose a large scale factor, *λ*, to increase the size of each Gaussian and smooth over smaller features. Gradient descent is iterated multiple times with *λ* decreasing multiple times. After the transformation, sample points are matched to reference points by minimizing distances between assigned pairs using an algorithm from [14]. The matching is not greedy, and neurons in the sample that are far from any neurons in the reference are not matched. A neuron at **x**_*i*_ is assigned a match *v*_*i*_ to indicate which neuron in the set **R** it was matched to. For example if **x**_*i*_ matched with **r**_*j*_ when **X** is registered to **R**, then *v*_*i*_ = *j*. If **x**_*i*_ has no match in **R**, then *v*_*i*_ = ∅.

The modified non-rigid point-set registration algorithm described above allows us to compare one constellation of neurons to another. In principle, neuron tracking could be achieved by registering the constellation of neurons at each time-volume to a single common reference. That approach is susceptible to failures in non-rigid point-set registration. Non-rigid point-set registration works well when the conformation of the animal in the sample and the reference are similar, but it is unreliable when there are large deformations between the sample and the reference, as happens with some regularity in our recordings. In addition, this approach is especially sensitive to any errors in segmentation, especially in the reference. An alternative approach would be to sequentially register neurons in each time volume to the next time-volume. This approach, however, accumulates even small errors and quickly becomes unreliable. Instead of either of those approaches, we use registration to compare the constellation of neurons at each time volume to a set of reference time-volumes that span a representative space of brain conformations (Fig 4b), as described below.

The constellation of neurons at a particular time in our recording is given by **X**_*t*_, and the position of the *i*-th neuron at time *t* is denoted by **x**_*i*,*t*_. We select a set of *K* reference constellations, each from a different time volume **X**_*t*_ in our recording, so as to achieve a representative sampling of the many different possible brain conformations the animal can attain. These *K* reference volumes are denoted by {**R**^1^, **R**^2^, **R**^3^,…,**R**^*K*^}. We use 300 volumes spaced evenly through time as our reference constellations. Each **X**_*t*_ is separately matched with each of the references, and each neuron in the sample, **x**_*i*,*t*_, gets a set of matches $v_{i,t}=\left\{v_{i,t}^{1},v_{i,t}^{2},v_{i,t}^{3},..v_{i,t}^{K}\right\}$, one match for each of the *K* references. This set of matches is a feature vector which we call a Neuron Registration Vector. It describes the neuron’s location in relation to its neighbors when compared with the set of references. This vector can be used to identify neurons across different times.

We find that 300 reference volumes creates feature vectors that are sufficiently robust to identify neurons in our recordings. What determines the optimal number of reference volumes? As long as the reference volumes contain a representative sample of the space of brain conformation occupied during our recordings, the number of reference volumes needed to create a robust feature vector depends only on the size of this conformation space. Because the conformation space of a real brain in physiological conditions is finite, there exists some number of reference volumes beyond which adding more reference volumes provides no additional information. Crucially, the worm brain seems to explore this finite conformation space quickly relative to the time scales of our recordings. As a result, the number of required reference volumes should not depend on recording length, at least for the minutes-long timescales that we consider here.

### Clustering registration vectors

The neuron registration vector provides information about that neuron’s position relative to its neighbors, and how that relative position compares with many other reference volumes. A neuron with a particular identity will match similarly to the set of reference volumes and thus that neuron will have similar neuron registration vectors over time. Clustering similar registration vectors allows for the identification of that particular neuron across time (Fig 4c and 4d).

To illustrate the motivation for clustering, consider a neuron with identity *s* that is found at different times in two sample constellations ***X***_1_ and ***X***_2_. When ***X***_1_ and ***X***_2_ have similar deformations, the neuron *s* from both constellations will be assigned the same set of matches when registered to the set of reference constellations, and as a result the corresponding neuron registration vectors **v**_1_ and **v**_2_ will be identical. This is true even if the registration algorithm itself fails to correctly match neuron *s* in the sample to its true neuron *s* in the reference. As the deformations separating ***X***_1_ and ***X***_2_ become larger, the distance between the feature vectors **v**_1_ and **v**_2_ also becomes larger. This is because the two samples will be matched to different neurons in some of the reference volumes as each sample is more likely to register poorly with references that are far from it in the space of deformations.

Crucially, the reference volumes consist of instances of the animal in many different deformation states. So while errors in registering some samples will exist for certain references, they do not persist across all references, and thus do not effect the entire feature vector. For the biologically relevant deformations that we observe, the distance between **v**_1_ and **v**_2_ will be smaller if both are derived from neuron *s* than compared to the distance between **v**_1_ and **v**_2_ if they were derived from *s* and another neuron. We can therefore cluster the feature vectors to produce groups that consist of the same neuron found at many different time points.

The goal of clustering is to assign each neuron at each volume to a cluster representing that neuron’s identity. Clustering is performed on the list of neuron registration vectors from all neurons at all times, {**v**_*i*,*t*_}. Each match in the vector, $v_{i,t}^{k}$, is represented as a binary vector of 0s with a 1 at the $v_{i}^{k-\text{th}}$ position. The size of the vector is equal to the number of neurons in **R**^*k*^. The feature vector {**v**_*i*,*t*_} is the concatenation of all of the binary vectors from all matches to the *K* reference constellations.

For computational efficiency, a two-step process was used to perform the clustering: First agglomerative hierarchical clustering was used on the neurons from an initial subset of volumes to define the clusters. Next, neurons from all volumes at all times were assigned to the nearest cluster as defined by correlation distance to the clusters’ center of mass. Assignments were made in such a way so as to ensure that a given cluster is assigned to at most one neuron per volume. Details of this clustering approach are described in the methods. Each cluster is given a label {*S*_1_, *S*_2_, *S*_3_,…} which uniquely identifies a single neuron over time, and each neuron at each time **x**_*i*, *t*_ is given an identifier *s*_*i*, *t*_ corresponding to the cluster to which that neuron-time belongs. Neurons that are not classified into one of these clusters are removed because they are likely artifactual or represent a neuron that is segmented too poorly for inclusion.

### Correcting errors in tracking and segmentation

**Fig 5:**
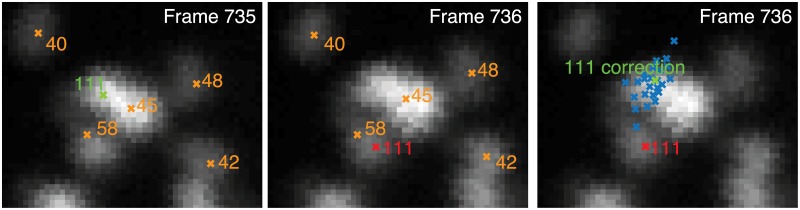
Example of consensus voting to correct a misidentified neuron. In volume 735, neuron #111 is found successfully and is indicated in green. In volume 736, however, the neuron is misidentified, shown in red. During the correction phase, all other time points vote for what the position of neuron #111 should based on a thin-plate spline deformation. A sample of votes are shown (blue ‘x’). Since the initial estimate of the position is far from the majority of consensus votes, a corrected position is assigned to be the centroid of the votes weighted by image intensity. This process is repeated to correct any errors for every neuron at every time.

Neuron Registration Vector Encoding successfully identifies segmented neurons consistently across time. A transient segmentation error, however, would necessarily lead to missing or misidentified neurons. To identify and correct for missing and misidentified neurons, we check each neuron’s locations and fill in missing neurons using a consensus comparison and interpolation in a TPS deformed space. For each neuron identifier *s* and time *t*^⋆^, we use all other point-sets, {**X**_*t*_} to guess what that neuron’s location might be. This is done by finding the TPS transformation, *u*_*t*→*t*^⋆^_: **X**_*t*_ ↦ **X**_*t*^⋆^_, that maps the identified points from **X**_*t*_ to the corresponding points in **X**_*t*^⋆^_ excluding the point *s*. Since the correspondences between neurons has already been determined, *u*_*t*→*t*^⋆^_ can be found by solving for the parameters from the TPS equation (see methods). The position estimate is then given by *u*_*t*→*t*^⋆^_ [**x**_*i*,*t*_] with *i* selected such that *s_i,t_* = *s*. This results in a set of points representing the set of predicted locations of the neuron at time *t*^⋆^ as inferred from the other volumes. When a neuron identifier is missing for a given time, the position of that neuron *s* is inferred by consensus. Namely, correct location is deemed to be the centroid of the set of inferred locations weighted by the underlying image intensity. This weighted centroid is also used if the current identified location of the neuron *s* has a distance greater than 3 standard deviations away from the centroid of the set of locations inferred from the other volumes, implying that an error may have occurred in that neuron’s classification. This is shown in Fig 5, where neuron 111 is correctly identified in volume 735, but the the label for neuron 111 is incorrectly located in volume 736. In that case the weighted centroid from consensus voting was used.

### Comparison with manually annotated data

To assess the accuracy of the Neuron Registration Vector Encoding pipeline, we applied our automated tracking system to a 4 minute recording of whole brain activity in a moving *C. elegans* that had previously been hand annotated and published [10]. A custom Matlab GUI was used for manually identifying and tracking neurons. Nine researchers collectively annotated 70 neurons from each of the 1519 volumes in the 4 minute video. This is much less than the 181 neurons predicted to be found in the head [26]. The discrepancy is likely caused by a combination of imaging conditions and human nature. The short exposure time of our recordings makes it hard to resolve dim neurons, and the relatively long recordings tend to cause photobleaching which make the neurons even dimmer. Additionally, human researchers naturally tend to select only those neurons that are brightest and are most unambiguous for annotation, and tend to skip dim neurons or those neurons that are most densely clustered.

**Fig 6:**
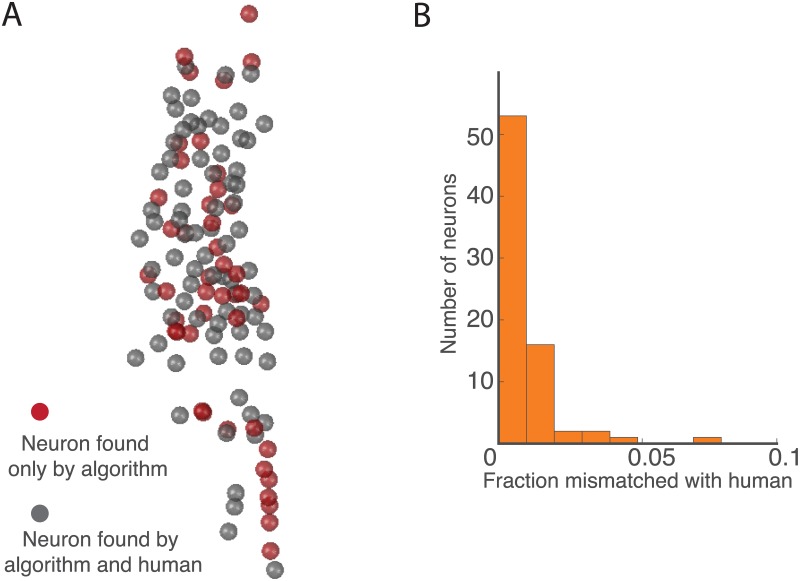
Comparison of the automated Neuron Registration Vector Encoding algorithm with manual human annotation. A previously published 4 minute recording of calcium activity (strain AML14) was annotated by hand, [10]. (A) Spheres show position of neurons that were detected by the automated algorithm. Grey indicates a neuron detected by both the algorithm and the human. All neurons detected by the human were also detected by the algorithm (70 neurons). Red indicates neurons that were missed by the human and detected only by the algorithm (49 neurons). (B) Histogram showing number of neurons that were mismatched for a given fraction of time-volumes when comparing automated and manual approaches. Only those neurons that were consistently found by both algorithm and human were considered. An automatically identified neuron was deemed correctly matched for a given time-volume if it was paired with the correct corresponding manual neuron.

We compared human annotations to our automated analysis in this same dataset. We performed the entire pipeline including detecting centerlines, worm straightening, segmentation, and neuron registration vector encoding and clustering, and correction. Automated tracking detected 119 neurons from the video compared to 70 from the human. In each volume, we paired the automatically tracked neurons with those found by manual detection by finding the closest matches in the unstraightened coordinate system. A neuron was perfectly tracked if it matched with the same manual neuron at all times. Tracking errors were flagged when a neuron matched with a manual neuron that was different than the one it matched with most often. The locations of the detected neurons are shown in Fig 6A. Only one neuron was incorrectly identified for more than 5% of the time volumes (Fig 6B). The locations of neurons and the corresponding error rates are shown in Fig 6B. Neurons that were detected by the algorithm but not annotated manually are shown in gray. Upon further inspection, it was noted that some of the mismatches between our method and the manual annotation were due to human errors in the manual annotation, meaning the algorithm is able to correct humans on some occasions.

GCaMP6s fluorescent intensity is ultimately the measurement of interest and this can be easily extracted from the tracks of the neuron locations across time. The pixels within an approximate 2 *μm* radius sphere around each point are used to calculate the average fluorescent intensity of a neuron in both the red RFP and green GCaMP6s channels at each time. This encompasses regions of the cell body, but excludes the neuron’s processes. The pixels within this sphere of interest are identified in the straightened RFP volume, but the intensity values are found by looking-up corresponding pixels in the unstraightened coordinate system in the original red- and green-channel images, respectively. We use the calcium-insensitive RFP signal to account for noise sources common to both the GCaMP6s and the RFP channel [10]. These include, for example, apparent changes in intensity due to focus, motion blur, changes in local fluorophore density arising from brain deformation and apparent changes in intensity due to inhomogeneities in substrate material. We measure neural activity as a fold change over baseline of the ratio of GCaMP6s to RFP intensity,

$$\begin{array}{ccc} \text{Activity}=\frac{\Delta R}{R_{0}}=\frac{R-R_{0}}{R_{0}}, & & \;R=\frac{I_{\text{GCaMP}6s}}{I_{\text{RFP}}}. \end{array}$$ (3)

**Fig 7:**
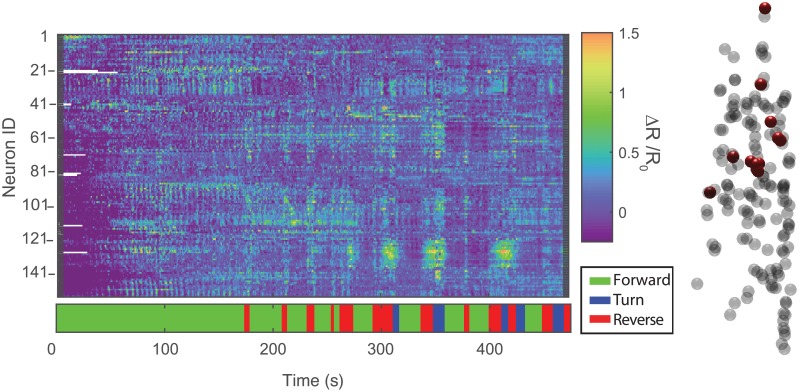
Calcium activity traces. Neural activity traces from 156 neurons in the brain a *C. elegans* as it freely moves on an agarose plate for 8 minutes (strain AML32). The neural activity is expressed as a fold change over baseline of the ratio of GCaMP6s to RFP for each neuron. The behavior is indicated in the ethogram. On the right is the locations of all of the detected neurons (the head of the worm is towards the top of the page). The neurons that have significant correlation with reverse locomotion are indicated in red. White gaps indicate instances where neurons failed to segment. This is a newly acquired recording, different from that in Fig 6.

The baseline for each neuron, *R*_0_, is defined as the 20th percentile value of the ratio *R* for that neuron. Fig 7 shows calcium imaging traces extracted from new whole-brain recordings using the registration vector pipeline. 156 neurons were tracked for approximately 8 minutes as the worm moves. Many neurons show clear correlation with reversal behaviors in the worm.

## Discussion

The Neuron Registration Vector Encoding method presented here is able to process longer recordings and locate more neurons with less human input compared to previous examples of whole-brain imaging in freely moving *C. elegans* [10]. Fully automated image processing means that we are no longer limited by the human labor required for manual annotation. In new recordings presented here, we are able to observe 156 of the expected 181 neurons, much larger than the approximately 80 observed in previous work from our lab and others [10, 11]. By automating tracking and segmentation, this relieves one of the major bottlenecks to analyzing longer recordings.

The neuron registration vector encoding algorithm primarily relies on the local coherence of the motion of the neurons. It permits large deformations of the worm’s centerline so long as deformations around the centerline remain modest. Crucially, the algorithm’s time-independent approach allows it to tolerate large motion between consecutive time-volumes. These properties make it well suited for our neural recordings of *C. elegans* and we suspect that our approach would be applicable to tracking neurons in moving and deforming brains from other organisms as well.

Certain classes of recordings, however, would not be well suited for Neuron Registration Vector Encoding and Clustering. The approach will fail when the local coherence of neuron motion breaks down. For example, if one neuron were to completely swap locations with another neuron relative to its surroundings, registration would not detect the switch and our method would fail. In this case a time-dependent tracking approach may perform better.

In addition, proper clustering of the feature vectors requires the animal’s brain to explore a contiguous region of deformation space. For example, if a hypothetical brain were only ever to occupy two distinct conformations that are different enough that registration is not reliable between these two conformation states, the algorithm would fail to cluster feature vectors from the same neuron across the two states. To effectively identify the neurons in these two conformations, the animal’s brain must sample many conformations in between those two states. This way, discrepancies in registration arise gradually and the resulting feature vectors occupy a continuous region in the space of possible feature vectors. Note that a similar requirement would necessarily apply to any time-dependent tracking algorithm as well.

We suspect that brain recordings from most species of interest meet these two requirements: namely neuron motion will have local coherence and the brain will explore a contiguous region of deformation space. Where these conditions are satisfied, we expect registration vector encoding to work well. Tracking in *C. elegans* is especially challenging because the entire brain undergoes large deformations as the animal bends. In most other organisms like zebrafish and *Drosophila*, brains are contained within a skull or exoskeleton and relative motion of the neurons is small. In those organisms, fluctuations in neuron positions take the form of rigid global transformations as the animal moves, or local non-linear deformations due to motion of blood vessels. We expect that this approach will be applicable there as well.

## Methods

### Strains

Transgenic worms were cultivated on nematode growth medium (NGM) plates with OP50 bacteria. Strain AML32 (wtfIs5[P*rab-3*::NLS::GCaMP6s; P*rab-3*::NLS::tagRFP]) was generated by UV irradiating animals of strain AML14 (wtfEx4[P*rab-3*::NLS::GCaMP6s; P*rab-3*::NLS::tagRFP]) [10] and outcrossing twice.

### Imaging *C. elegans*

Imaging is performed as described in Nguyen et al [10]. The worm is placed between an agarose slab and a large glass coverslip. The coverslip is held up by 0.006” plastic shims in order to reduce the amount of pressure on the worm from the glass, and mineral oil is spread over the worm to better match refractive indices in the space between the coverglass and the worm. The dark field image is used to extract the animal’s centerline while the fluorescent image is used for tracking the worm’s brain. Only the head of the worm is illuminated by the fluorescent excitation light and can be observed in the low magnification fluorescent image.

The two low magnification videos and the RFP and GCaMP6 high magnification videos are aligned by imaging a slide of 4 *μm* “Tetraspeck” beads (ThermoFisher) that emit light in both red and green channels. We manually or automatically locate the beads from calibration images and use the bead positions to find affine transformations between each camera’s coordinate system. The affine parameters are found using a least squares fit on the coordinates of the beads in the image.

### Thin plate spline transformations

Thin plate spline (TPS) transformations play an important role in error correcting and are also critical for the point set registration algorithm [24]. Given a set of *n* initial control points **X** = {**x**_*i*_}, and the set of transformed points, *u*[**X**], the TPS transformation *u* can be written as *u*[**X**] = **WU**(**X**) + **AX** + **t**. The affine portion of the transformation is **AX** + **t**, while **WU**(**X**) is the non-linear part of the transformation from TPS. **U**(**X**) is an *n* × *n* vector with $U_{i,j}=\frac{1}{\left\|x_{j}-x_{i}\right\|}$ and **W** is a 3 × *n* matrix. The elements of **W**, **A** and **t** are the parameters of the transformation *u*. These parameters are found in different ways dependant on context. During the error correction processing step, these parameters are fit by knowing both the the set of control points **X** and the location of the transformed points *u*[**X**]. In the context of the point set registration algorithm, *u*[**X**] incurs an energy penalty for deforming space given by *E*_Deformation_(*u*) = trace(**WUW**^**T**^) [24]. This cost is used in Eq 2 to determine the total energy of the transformation. Gradient descent is then used to determine the optimal TPS transformation parameters by minimizing the total energy of the transformation.

### Clustering

Clustering is performed in two steps: hierarchical clustering and neuron classification. We chose to perform hierarchical clustering only on an initial subset of 800 volumes because hierarchical clustering can become prohibitively computationally intensive for larger datasets. The correlation distance, 1 − corr(**v**_*m*_, **v**_*n*_), was used as the pairwise distance metric for clustering. Agglomerative hierarchical clustering was implemented using complete linkage with a distance cutoff of 0.9. Clusters which are smaller than 40% of the number of subset volumes were removed. After the clusters were defined via hierarchical clustering, we then performed neuron classification.

To classify neurons, we assigned neurons from every volume to the cluster with the nearest centroid. Only the best matched neuron in each volume is assigned to a cluster and only if the neuron is closer than some threshold distance, described below. If two or more neurons from a volume would otherwise be assigned to a single cluster, the closest neuron retains that classification and other neurons are unassigned. As a result, some putative neurons are not assigned to any cluster and at most one neuron per volume is assigned to any given cluster. The implementation of the algorithm is shown in Algorithm 1.

**Algorithm 1** Clustering the Neuron Registration Vectors

 1: **input:** Set of registration vectors *V* = {**v**_*i*,*t*_}

 2: **output:** Cluster assignments for each of the vectors in *V*

 3: **procedure** Cluster(*V*)

 4:  *S* = subset of *V*

 5:  *subset*_*assignments* = hierarchically cluster *S* with distance cutoff 0.9

 6:  *cluster*_*list* = unique(*subset*_*assignments*)

 7:  **for** each *cluster* in *cluster*_*list* **do**

 8:   **If** size(*cluster*) > 40% of volumes used **then**

 9:    *cluster*_*center* = average of *S* assigned to *cluster*

10:   **else**

11:    remove *cluster*

12:   **end if**

13:  **end for**

14:  compute *threshold* from *S*

15:  **for** each **v**_*i*,*t*_ in *V* **do**

16:   *d* = distances from **v**_*i*,*t*_ to *cluster*_*centers*

17:   **if** any(*d* < *threshold*) **then**

18:    assign **v**_*i*,*t*_ to closest cluster

19:   **end if**

20:  **end for**

21:  **for** each volume in the recording **do**

22:   **for** each *cluster* in *cluster*_*list* **do**

23:    **if** multiple **v**_*i*,*t*_ from volume assigned to cluster **then**

24:     unassign all **v**_*i*,*t*_ from the cluster except the closest one

25:    **end if**

26:   **end for**

27:  **end for**

28: **end procedure**

The threshold distance to determine whether a neuron is assigned to a cluster is calculated using a statistical analysis of the clusters generated by the initial hierarchical clustering so as to discriminate between neurons that are likely correctly or incorrectly assigned. The threshold is calculated as follows: For each neuron assigned during the initial clustering, we collect the distance between that neuron and the center of the cluster it was assigned to. The distribution of these distances is the “correctly assigned” distribution. In contrast, the null distribution is found by collecting the distances between each neuron and all clusters to which that neuron is not assigned. The threshold distance is set to be the largest distance for which a distance is more likely to be found in the “correctly assigned” distribution than the null distribution.

### Algorithm implementation

The analysis was performed on Princeton University’s high-performance scientific computing cluster, “Della” primarily consisting of 240 nodes and 4288 cores, each with 2.4 GHz processors. Jobs were run on up to 200 cores simultaneously. Timing information for the steps listed in Fig 1 are described below and summarized in Table 1.

**Table 1:** Breakdown of computation time and scalings for Neuron Registration Vector Encoding pipeline. *n*_frames_ is the total number of low magnification images used to detect centerlines, *n*_vol_ is the total number of volumes in the recording, *n*_ref_ is the number of reference volumes used for creating feature vectors, *n*_neurons_ is the total number of neurons detected, and *n*_subset_ is the number of neurons in the subset of volumes used for initial clustering.

Analysis Step		Computation	Approx. % of real time	Computational time scales as
Centerline Detection		Linear	4	*O*(*n*_frames_)
Worm Straightening		Parallel	10	*O*(*n*_vol_)
Segmentation				
Registration Vector Encoding		Parallel	80	*O*(*n*_vol_ × *n*_ref_)
Clustering	Hierarchical	Linear	2	$O(n_{\text{subset}}^{2}\times n_{\text{neurons}}^{2})$
	Classification	Linear	0	*O*(*n*_vol_ × *n*_neurons_)
Error Correction		Parallel	4	up to $O(n_{\text{vol}}^{2}\times n_{\text{neurons}})$

#### Centerline detection

Centerlines in each image are calculated using information from the previous centerline and as a result must be computed linearly. Total computational time for centerline detection scales linearly with recording length. Specifically, centerlines must be fit for every frame of the low magnification video so the computation time scales as *O*(*n*_frames_).

#### Worm straighteningand and *segmentation*

Straightening and segmentation are parallelized over each volume. Total computation time for worm straightening and segmentation scales as *O*(*n*_vol_), with each volume taking ∼20 seconds on a single core.

#### Registration vector construction

Registration vector construction is the most computationally intensive part of the algorithm. The vectors are created by performing non-rigid point-set registration between all sample volumes and all reference volumes. Total computation time scales as *O*(*n*_vol_ × *n*_ref_) with the bulk of the computational time consumed by gradient descent during point-set registration. The gradient descent for matching a single sample volume to a single reference on one processing core takes ∼15s. This step is parallelized over each volume.

#### Clustering

Clustering is broken into two subparts, hierarchical clustering and classification. Clustering is overall fast compared to point-set registration. Computation time for hierarchical clustering scales quadratically with the number of neurons in the worm times the size of the initial set of volumes used to define the clusters. This can become prohibitively slow if we increase the number of volumes used for hierarchical clustering as the length of the recordings increase. Thus, we fix the number of volumes used for initial hierarchical clustering to an arbitrary size (e.g. 800 volumes) regardless of the length of the recording. Thus, the clustering time scales as $O(n_{\text{subset}}^{2}\times n_{\text{neurons}}^{2})$. After the clusters are defined via hierarchical clustering in the first subpart, all neurons can then be quickly classified into clusters with negligible computational time in the second subpart.

#### Error correction

Error correction is parallelized over each neuron, with each neuron checked in every volume. The error checking in each volume is done by comparing the point-set to all other point-sets using a thin-plate spline transformation. This operation is fast compared to the registration vector construction step because here the correspondences between the point sets are already known. Computation time for this process scales as $O(n_{\text{vol}}^{2}\times n_{\text{neurons}})$. We chose to compare each neuron in each volume to all other volumes because these comparisons are relatively fast. However, we also observe that comparing each neuron with merely a subset of volumes seems to suffice without loss of performance. In that case the computation time for error correction would be *O*(*n*_vol_ × *n*_neurons_ × *n*_subset_), where *n*_subset_ is the number of volumes selected for comparison during error correcting.

An 8 minute recording of a moving animal has approximately 3000 volumes and 250 GB of raw imaging data and can be processed from start to finish on the university cluster in less than 40 hours. Major time reduction could be achieved by reducing the number of reference volumes used during Registration Vector Encoding.

All data used in this publication have been made publicly available at the IEEE DataPort repository (DOI:10.21227/H2901H) http://dx.doi.org/10.21227/H2901H. MATLAB code implementing our pipeline is available at https://github.com/leiferlab/NeRVEclustering.

## Supporting information

S1 Movie Example video for raw, straightened, and tracked data. Left: Raw video feed from high magnification RFP video. The imaging plane is scanning up and down through the volume of the worm’s brain. The recording is shown at 1/2× speed and the time elapsed is indicated in the bottom left. Middle: Maximum intensity projection of each volume is shown after Worm Centerline Tracking and Straightening. Right: Locations of neurons are shown at the end of the pipeline (after Neuron Registration Vector Encoding, Clustering and Error Correction). Each color represents a different tracked neuron. All neurons from the volume are shown overlaid on a raw image of the middle plane of each volume. Note a light flash used for time synchronization is visible around *t* = 13s.
(MP4)
